# In Older Adults the Antidepressant Effect of Repetitive Transcranial Magnetic Stimulation Is Similar but Occurs Later Than in Younger Adults

**DOI:** 10.3389/fnagi.2022.919734

**Published:** 2022-07-19

**Authors:** Gonçalo Cotovio, Aaron D. Boes, Daniel Z. Press, Albino J. Oliveira-Maia, Alvaro Pascual-Leone

**Affiliations:** ^1^Champalimaud Research and Clinical Centre, Champalimaud Foundation, Lisbon, Portugal; ^2^NOVA Medical School, NMS, Universidade Nova de Lisboa, Lisbon, Portugal; ^3^Department of Psychiatry and Mental Health, Centro Hospitalar de Lisboa Ocidental, Lisbon, Portugal; ^4^Department of Psychiatry, University of Iowa Carver College of Medicine, Iowa City, IA, United States; ^5^Department of Neurology, University of Iowa Carver College of Medicine, Iowa City, IA, United States; ^6^Department of Pediatrics, University of Iowa Carver College of Medicine, Iowa City, IA, United States; ^7^Division of Cognitive Neurology, Department of Neurology, Berenson-Allen Center for Noninvasive Brain Stimulation, Beth Israel Deaconess Medical Center, Harvard Medical School, Boston, MA, United States; ^8^Hinda and Arthur Marcus Institute for Aging Research, Deanna and Sidney Wolk Center for Aging Research, Hebrew SeniorLife, Boston, MA, United States; ^9^Department of Neurology, Harvard Medical School, Boston, MA, United States

**Keywords:** transcranial magnetic stimulation, depression, older adults, naturalistic study, efficacy

## Abstract

**Background:**

Treatment resistant depression is common in older adults and treatment is often complicated by medical comorbidities and polypharmacy. Repetitive transcranial magnetic stimulation (rTMS) is a treatment option for this group due to its favorable profile. However, early influential studies suggested that rTMS is less effective in older adults. This evidence remains controversial.

**Methods:**

Here, we evaluated the rTMS treatment outcomes in a large international multicenter naturalistic cohort of >500 patients comparing older vs. younger adults.

**Results:**

We show that older adults, while having similar antidepressant response to younger adults, respond more slowly, which may help to explain differences from earlier studies when the duration of a treatment course was shorter.

**Conclusions:**

Such evidence helps to resolve a long-standing controversy in treating older depressed patients with rTMS. Moreover, these findings provide an important data point in the call to revise policy decisions from major insurance providers that have unfairly excluded older adults.

## Introduction

Major Depressive Disorder (MDD) is a leading cause of disability worldwide, and commonly affects older adults (Beekman et al., 1995). Older adult patients are more likely to have treatment resistant depression (Little et al., 1998) and treatment is particularly challenging due to comorbidities and polypharmacy (Tedeschini et al., 2011; Kok and Reynolds, 2017). Brain stimulation strategies, namely repetitive transcranial magnetic stimulation (rTMS), have been considered an effective antidepressant treatment for those who do not respond or tolerate other treatment strategies (Mutz et al., 2019). This approach has also been supported in preclinical studies, with evidence of antidepressant-like effects in animal models (De Risio et al., 2020). Due to its favorable side-effects profile (Machii et al., 2006), lower likelihood of drug–drug interaction (George, 2010), and potential cognitive enhancing effects (Martin et al., 2017; Chou et al., 2019), rTMS is a particularly interesting antidepressant treatment modality for older patients with depression. However, clinical efficacy of rTMS in older patients has been controversial. In fact, while older age has been considered a predictor of poorer rTMS antidepressant efficacy (Fregni et al., 2006), a recent metanalysis of randomized controlled trials supported that rTMS is a clinically effective antidepressant treatment in older patients (Valiengo et al., 2022), and there are suggestions that rTMS has similar antidepressant efficacy in the older and younger patients (Conelea et al., 2017; Sackeim et al., 2020). However, evidence to support either of these views is still lacking. This still has important clinical implications since some major health insurance providers, mainly in USA, have policies that limit coverage of rTMS for older adults. Here, we aim to assess if rTMS antidepressant response differs in overall efficacy between the older and younger patients, and if the trajectory of response differs.

## Methods

We conducted an international multicenter naturalistic retrospective study, with data from adult patients treated with rTMS for a major depressive episode in the context of MDD or bipolar disorder type II, irrespective of medication status, at three different rTMS clinical centers: Berenson-Allen Center for Noninvasive Brain Stimulation (Boston, USA), University of Iowa Center for Noninvasive Brain Stimulation (Iowa City, USA), and Champalimaud Foundation Neuropsychiatry Unit (Lisbon, Portugal), from 2000 to 2021, in compliance with each site's local Internal Review Board (IRB) policies for analysis and publication of clinical data. All adult patients treated with DLPFC rTMS for depression at Boston and Iowa centers were considered for inclusion since informed consent for retrospective clinical data inclusion in the current analysis was exempted by local IRBs. For Lisbon center, only patients who signed informed consent were considered. Depression was considered as any major depressive episode in the context of either MDD or bipolar disorder type II, according to the Diagnostic and Statistical Manual of Mental Disorders (American Psychiatric Association, 1994, 2000, 2013). Patients were eligible if treated with Magstim, Neuronetics, or Magventure devices. Patients were excluded if age or motor threshold at first session could not be retrieved, if treatment device changed during treatment, if less than 10 sessions were conducted, or if average interval between consecutive sessions was ≥2.5 days. The following data were extracted from electronic clinical databases: age, sex, baseline (week 1), and weekly self-report depression severity scores (Beck Depression Inventory, BDI: Boston and Lisbon cohorts; Patient Health Questionnaire, PHQ-9: Iowa City cohort), stimulation parameters such as treatment device, stimulation side, and stimulation protocol (1, 10, 18, or 20 Hz rTMS; intermittent theta burst stimulation, iTBS), including the total number of pulses per treatment, and weekly resting motor threshold. Patients were considered younger when less than 65 years-old, and older when 65 years-old or older. Clinical response to rTMS was calculated as the percent reduction of self-reported depression severity scores at each measurement relative to baseline (week 1). Data for continuous measurements are presented as mean ± standard error of the mean (SEM). Patients were considered responders when a reduction of severity of at least 50% relative to baseline (week 1) was observed, with data presented as percent of patients (%).

For statistical analyses, continuous variables were analyzed using longitudinal mixed effects regression analyses, where the dependent variables were the percent reduction of self-reported depression severity scores, or the absolute difference from baseline in BDI or PHQ-9 scores. The independent variables in the longitudinal mixed effects regression models included week of treatment, age group and their interaction term. *Post-hoc* exploratory unpaired two sample *t*-tests at each time point were performed and corrected for multiple comparisons using False Discovery Rate (FDR) of 0.05, according to Benjamini and Hochberg (1995). Additional longitudinal mixed effects regression analyses were performed, similar to those mentioned above, controlling for potential confounding effects of sex, TMS device, stimulation side, stimulation protocol (1, 10, 18, or 20 Hz rTMS or iTBS), treatment site, year of treatment, and total number of pulses per treatment, as well as baseline self-report depression severity when appropriate. Differences in response status between age groups were analyzed using chi-squared tests and corrected for multiple comparisons as above. All data were analyzed using StataCorp. 2017. Stata Statistical Software: Release 15. College Station, TX: StataCorp LLC.

## Results

**Figure 1:**
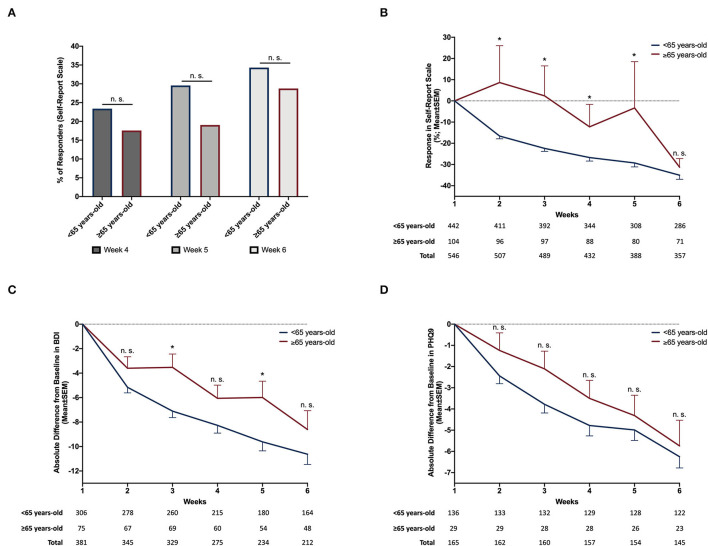
Differences in antidepressant response across rTMS treatment between <65 and ≥65 years-old patients. When comparing the percentage of responders between <65 years-old and ≥65 years-old group using a chi-squared test, no differences were observed in any of the tested time points: 4th week−23.4 vs. 17.6%, *p* = 0.2|5th week−29.6 vs. 19.1%, *p* = 0.06|6th week−34.3% vs. 28.8%, *p* = 0.3 **(A)**. When analyzed using longitudinal mixed effects regression model, % change of depression severity in self-report scale was found to decrease across time (ß = −7.8 ± 0.8, *p* < 0.0001). No differences were observed between the two age groups (ß = 2.8 ± 3.6, *p* = 0.4) but we found a significant interaction between time and age group (ß = 5.5 ± 1.9, *p* = 0.003). *Post-hoc t*-tests revealed larger decreases of depression severity in <65-years-old when compared to ≥65 years-old until week 5 **(B)**. When considering absolute difference of BDI scores relative to baseline (week 1), we also observed that depression severity decreased across time (ß = −2.8 ± 0.2, *p* < 0.0001) as well as a significant effect of age group (ß = −1.3 ± 0.6, *p* < 0.04) and interaction (ß = 1.4 ± 0.4, *p* < 0.0001). *Post-hoc t*-tests revealed larger decrease of depression severity in <65-years-old only in week 3 and 5 **(C)**. When considering absolute difference of PHQ9 scores from baseline (week 1), there was a significant effect of time (ß = −1.1 ± 0.1, *p* < 0.0001), but no effect of age group (ß = 0.8 ± 0.5, *p* < 0.1) or interaction (ß = 0.002 ± 0.2, *p* < 1.0). No differences were found in *post-hoc* comparisons between the two age groups **(D)**. For plots **(B–D)**, while total sample mostly drops across time due to attrition, there are increases in some instances due to reductions in missing values. *p < 0.05, *post-hoc* exploratory *t*-tests corrected for multiple comparison using False Discovery Rate (FDR) of 0.05, according to Benjamini and Hochberg (1995); BDI, Beck Depression Inventory; n.s., non-significant; PHQ9, Patient Health Questionnaire; SEM, Standard Error of the Mean.

Data were collected and analyzed from a total of 546 patients, 58.6% of whom were women, including 442 <65 years-old and 104 ≥65 years-old. Mean (±SEM) baseline BDI and PHQ9 were 29.3 (±0.6) and 17.8 (±0.4), respectively (see Table 1 for further details). At baseline (week 1), when comparing <65 and ≥65 years-old age groups, depression severity in younger adults was higher, when assessed with BDI, and similar, when assessed with PHQ-9, when compared to older adults (baseline BDI: 29.9 ± 0.6 vs. 26.9 ± 1.3, *p* = 0.03; baseline PHQ-9: 18.0 ± 0.4 vs. 17.0 ± 1.0, *p* = 0.3). We did not find a difference between the two age groups regarding the percentage of responders (Figure 1A) when assessed at the most common TMS clinical trial endpoints, i.e., weeks 4 (*N* = 372 vs. 91), 5 (*N* = 318 vs. 84), and 6 (N = 303 vs. 80). Regarding trajectory of response to treatment, measured according to % reduction of depression severity in self-report scales, we found that severity decreased across time (ß = −7.8 ± 0.8, *p* < 0.0001; Figure 1B; Table 2) during the rTMS treatment. While no differences were observed between the two age groups, there was a significant interaction between time and age group (ß = 5.5 ± 1.9, *p* = 0.003), with depression severity reducing more rapidly among younger than in older adults. Similar results were obtained when controlling for potential confounding effects of sex, TMS device, stimulation side, stimulation protocol (1, 10, 18, or 20 Hz rTMS or iTBS), treatment site, year of treatment, and total number of pulses per treatment (Table 2). The *post-hoc* comparisons between the two age groups revealed differences in every week of assessment, favoring larger decrease of depression severity in younger when compared to older patients, until week 5, but no differences observed in week 6. A somewhat similar pattern was also found when considering absolute difference in BDI scores from baseline (Figure 1C; Table 2), with a significant reduction of depression severity across rTMS treatment (ß = −2.8 ± 0.2, *p* < 0.0001), and significant effects of age group (ß = −1.3 ± 0.6, *p* = 0.04) and the interaction between time and age (ß = 1.4 ± 0.4, *p* < 0.0001). When controlling for the potential confounders mentioned above, as well as baseline BDI, similar effects were obtained for time and interaction, but without a significant effect for age group (Table 2). The *post-hoc* comparisons between the two age groups revealed differences only in weeks 3 and 5 of assessment, with larger decreases of depression severity in younger patients. In analyses of absolute difference from baseline with PHQ-9 (Figure 1D; Table 2), used alternatively to BDI in one of the centers, there was a significant effect of time (ß = −1.1 ± 0.1, *p* < 0.0001), but no effect of age group or interaction between both time and age group. Similar effects were observed when controlling for confounders, including baseline PHQ-9 (Table 2), with no differences found between the two age groups in *post-hoc* comparisons.

**Table 1:** Demographic and clinical characteristics of study sample.

**Characteristic**	**Total sample** **(*N* = 546)**	**Study centers**			**Age group**	
	**Mean ±SEM or %**	**Boston** **(*N* = 346)**	**Iowa City** **(*N* = 165)**	**Lisbon** **(*N* = 35)**	** <65** **(*N* = 442)**	**≥65** **(*N* = 104)**
		**Mean ±SEM** **or %**	**Mean ±SEM** **or %**	**Mean ±SEM** **or %**	**Mean ±SEM** **or %**	**Mean ±SEM** **or %**
**Age group**					N.A.	N.A.
<65 years-old	80.9	79.8	82.4	85.7		
≥65 years-old	19.1	20.2	17.6	14.3		
**Age**	49.2 ± 0.7	50.1 ± 0.8	47.6 ± 1.3	48.6 ± 2.8	44.3 ± 0.6	70.4 ± 0.4
**Sex (% female)**	58.6	59.4	58.5	51.4	59.8	53.9
**Depression**						
Unipolar	92.9	88.8	100.0	80.0	92.8	93.2
Bipolar	7.1	11.2	0.0	20.0	7.2	6.8
**Stimulation side (% left)**	91.3	89.1	96.4	88.6	91.6	90.2
**Study protocol**						
1 Hz rTMS	8.4	10.8	3.6	11.4	8.3	9.0
10 Hz rTMS	41.2	44.8	43.6	0.0	38.9	51.7
18 Hz rTMS	0.6	1.0	0.0	0.0	0.3	2.3
20 Hz rTMS	25.6	43.4	0.0	0.0	27.6	16.9
iTBS	4.2	0.0	52.7	88.6	25.1	20.2
**Stimulation device**						
Magstim	46.7	73.7	0.0	0.0	46.6	47.1
Neuronetics	16.7	26.3	0.0	0.0	15.8	20.2
Magventure	36.6	0.0	100.0	100.0	37.6	32.7
**Pulses per treatment**	1,929.0 ± 44.1	2,231.0 ± 40.9	1,669.1 ± 91.9	668.6 ± 32.7	1,885.2 ± 48.5	2,125.4 ± 103.6
**Baseline HAMD^a^**	21.0 ± 0.4	21.0 ± 0.4	N.A.	N.A.	22.1 ± 0.5	21.4 ± 0.7
**Baseline MADRS^a^**	28.7 ± 0.5	N.A.	29.4 ± 0.5	25.4 ± 1.5	28.6 ± 0.6	29.1 ± 1.4
**Baseline BDI^b^**	29.3 ± 0.6	29.5 ± 0.6	N.A.	28.1 ± 1.9	29.9 ± 0.6	26.9 ± 1.3
**Baseline PHQ9^b^**	17.8 ± 0.4	N.A.	17.8 ± 0.4	N.A.	18.0 ± 0.4	17.0 ± 1.0

*BDI, Beck Depression Inventory; HAMD, Hamilton Depression Rating Scale; Hz, Hertz; iTBS, Intermittent Theta Burst Stimulation; MADRS, Montgomery–Åsberg depression rating scale; N, number of subjects; N.A., non-applicable; PHQ9, Patient Health Questionnaire; rTMS, repetitive transcranial magnetic stimulation; SEM, standard error of the mean*.

a *HAMD was used to assess clinitian-rated depression severity in Boston while MADRS was used in Iowa city and Lisbon*.

b *BDI was used to assess self-reported depression severity in Boston and Lisbon while PHQ9 was used in Iowa city*.

**Table 2:** Statistical models of antidepressant response across transcranial magnetic stimulation treatment.

**Independent variable**	**Percentage reduction** **of self-report depression severity**				**Absolute difference of** **BDI scores from baseline**				**Absolute difference of** **PHQ9 scores from baseline**			
	**Non-adjusted**		**Adjusted^a^**		**Non-adjusted**		**Adjusted^a^**		**Non-adjusted**		**Adjusted^a^**	
	**ß ±SEM**	***p*** **-value**	**ß ±SEM**	***p*** **-value**	**ß ±SEM**	***p*** **-value**	**ß ±SEM**	***p*** **-value**	**ß ±SEM**	***p*** **-value**	**ß ±SEM**	***p*** **-value**
**Age group**	2.8 ± 3.6	0.4	3.6 ± 4.1	0.4	−1.3 ± 0.6	0.04	−1.4 ± 0.7	0.06	0.8 ± 0.5	0.1	0.7 ± 0.5	0.2
**Week**	−7.8 ± 0.8	<0.0001	−7.8 ± 0.9	<0.0001	−2.8 ± 0.2	<0.0001	−2.9 ± 0.2	<0.0001	−1.1 ± 0.1	<0.0001	−1.1 ± 0.1	<0.0001
**Age group x Week**	5.5 ± 1.9	0.003	6.0 ± 2.1	0.005	1.4 ± 0.4	<0.0001	1.4 ± 0.4	0.001	0.002 ± 0.2	1.0	−0.0007 ± 0.2	1.0
**Sex (Ref.: Female)**	N.A.	N.A.	−1.4 ± 3.2	0.7	N.A.	N.A.	0.3 ± 0.4	0.4	N.A.	N.A.	−0.1 ± 0.4	0.8
**TMS Device (Ref.: Magstim)**	N.A.	N.A.			N.A.	N.A.	–		N.A.	N.A.	N.A.	N.A.
Neuronetics			−2.4 ± 4.6	0.6			0.06 ± 0.5	0.9				
Magventure			−5.5 ± 8.6	0.5			1.2 ± 2.0	0.6				
**Stimulation side (Ref.: Right)**	N.A.	N.A.	6.3 ± 23.7	0.8	N.A.	N.A.	1.9 ± 2.6	0.5	N.A.	N.A.	0.5 ± 1.0	0.6
**Stim. protocol (Ref.: 1Hz rTMS)**	N.A.	N.A.			N.A.	N.A.			N.A.	N.A.		
10 Hz rTMS			−2.3 ± 56.4	0.9			−1.8 ± 2.6	0.5			−0.9 ± 0.5	0.07
18 Hz rTMS			−6.6 ± 34.2	0.8			−1.8 ± 3.4	0.6			N.A.	N.A.
20 Hz rTMS			−8.2 ± 24.2	0.7			−2.1 ± 2.7	0.4			N.A.	N.A.
iTBS			−5.4 ± 39.3	0.9			−3.6 ± 3.2	0.3			Omitted^d^	Omittedd^d^
**Site (Ref.: Boston)**	N.A.	N.A.			N.A.	N.A.			N.A.	N.A.	N.A.	N.A.
**Iowa City**			2.0 ± 6.5	0.8			N.A.	N.A.				
**Lisbon**			Omitted^b^	Omitted^b^			Omitted^b^	Omitted^b^				
**Year of treatment**	N.A.	N.A.	−0.2 ± 0.8	0.8	N.A.	N.A.	−0.004 ± 0.09	1.0	N.A.	N.A.	−0.2 ± 0.2	0.3
**Pulses per treatment**	N.A.	N.A.	−0.002 ± 0.04	1.0	N.A.	N.A.	Omitted^c^	Omitted^c^	N.A.	N.A.	Omitted^e^	Omitted^e^
**Baseline BDI**	N.A.	N.A.	N.A.	N.A.	N.A.	N.A.	−0.03 ± 0.02	0.09	N.A.	N.A.	N.A.	N.A.
**Baseline PHQ9**	N.A.	N.A.	N.A.	N.A.	N.A.	N.A.	N.A.	N.A.	N.A.	N.A.	−0.07 ± 0.03	0.06

*BDI, Beck Depression Inventory; Hz, Hertz; iTBS, Intermittent Theta Burst Stimulation; N.A., not applicable; PHQ9, Patient Health Questionnaire; Ref., Reference; rTMS, repetitive transcranial magnetic stimulation; SEM, standard error of the mean; Stim., stimulation; TMS, transcranial magnetic stimulation*.

a *Models are adjusted for potential confounding effects of sex, TMS device, stimulation side, stimulation protocol (1Hz, 10Hz, 18Hz, or 20Hz rTMS or iTBS), treatment site, year of treatment, total number of pulses per treatment*.

b *Omitted because of collinearity with TMS device*.

c *Omitted because of collinearity with stimulation protocol and study center*.

d *Omitted because of collinearity with stimulation side*.

e *Omitted because of collinearity with stimulation protocol*.

## Discussion

These results provide evidence that rTMS has similar efficacy for treating depression in older and younger adults, as studied in a large naturalistic sample. However, the data suggest that the antidepressant response trajectory differs between these two groups, favoring a slower antidepressant response in older patients. Hence, our results suggest a discrepancy in TMS efficacy between age groups observed in the first weeks of treatment, but that is no longer significantly present when treatment is completed. In fact, this finding could justify why age was previously considered a poor predictor, since only 2 weeks of treatment were considered in that predictive analysis (Fregni et al., 2006). In fact, if we had only considered this shorter treatment period, treatment responses would be significantly different between older and younger patients, with amelioration of mean depression severity only in the younger group. However, when at least a full 6-week rTMS treatment was offered, no differences in treatment response were observed between the two age groups, suggesting that more sessions are needed in older adult patients to improve depression severity. These findings are consistent with our previously published work (Valiengo et al., 2022), where a meta-regression also suggested that more rTMS sessions were associated with enhanced antidepressant response in older adults. Other authors have previously suggested that improved efficacy of rTMS in older adults could be achieved with higher stimulation intensities, to overcome a greater distance from coil to brain, secondary to prefrontal atrophy in this population (Nahas et al., 2004). However, increasing stimulation intensity in the presence of atrophy can also lead to greater shunting of the induced current through the cerebrospinal fluid, and thus lower focality of the stimulation (Wagner et al., 2007). Thus, we argue for the potentially safer and more effective approach of allowing for a greater number of sessions in older adults, and hypothesize that in this population, longer rTMS courses, up to 8- or 10-weeks long, may be even more effective. This hypothesis, while supported by the greater antidepressant response at 6 relative to 4-weeks of rTMS treatment (O'Reardon et al., 2007), requires further empirical support.

Our study is not without limitations that should be considered. First, this study has a retrospective, naturalistic, and multicenter design. Accordingly, different socio-demographic or clinical variables, such as education, medication, and illness duration, were not available to collect or analyze, which may potentially limit the interpretation of the findings. While this study design can be regarded as a limitation, it also has some strengths. In fact, clinical research environments that enroll highly selected patients may lack external validity (Fagiolini et al., 2017). This is usually not the case with naturalistic studies, which may more closely reflect the general population of patients, and thus be more generalizable. Additionally, we have analyzed data from a wide range of time, from three different centers. This approach typically further improves the generalizability of results and, if bias occurs, it is most likely non-differential information/misclassification (Ahrens and Pigeot, 2014). Such type of bias favors the null hypothesis across analysis (Ahrens and Pigeot, 2014), which was not the case in the present study, where we found significant differences between age groups, namely in the first stages of treatment, even when adjusting the analysis for potential socio-demographic and clinical confounders available for analysis, and/or correcting for multiple comparisons. Finally, we have also repeated all statistical models, while adjusting for potential confounders, extracted or all available socio-demographic and clinical variables, and have confirmed results of the original models. Second, since we found close to significant differences in treatment response between age groups at week 5 (Figure 1A), it is possible that we may have lacked statistical power to detect significant differences in response rates between groups. Nevertheless, while the difference between % responders in younger and older adults was apparently of larger magnitude at 5 weeks, it still did not reach significance, albeit a reasonably large sample size. Furthermore, this possible difference was markedly diminished at 6 weeks. These findings support that clinical meaningful efficacy is equivalent at the end of treatment. We believe that our results provide important evidence for efficacy of TMS in older adults even in light of these potential limitations.

In conclusion, our results demonstrate that rTMS is an effective treatment for depression in older patients but that longer rTMS therapeutic courses of at least 6-weeks should be provided. This conclusion is of particular importance when considering that some major insurance providers, namely in the USA, have policies that preclude insurance coverage of rTMS for older adults or that make extension of the treatment course beyond 4 weeks dependent on the efficacy achieved up to that point. We expect that the evidence presented here will lead to a revision of such policies, to ensure maximal benefit for older adults who are in particular need of effective treatment option when afflicted by a depressive episode.

## Data Availability Statement

The datasets presented in this article are not readily available because it was generated from different participating centers. Requests to access the datasets should be directed to AJO-M (albino.maia@neuro.fchampalimaud.org) and/or AP-L (apleone@hsl.harvard.edu).

## Ethics Statement

These studies were reviewed and approved by the Champalimaud Foundation Ethics Committee and the Beth Israel Deaconess Medical Center and Iowa University local Institutional Review Boards (IRBs). Champalimaud Foundation patients/participants provided their written informed consent to participate in this study. Beth Israel Deaconess Medical Center and Iowa University IRBs exempted informed consent for retrospective clinical data.

## Author Contributions

GC, AB, DP, AJO-M, and AP-L conceived and designed the work, acquired the data, and analyzed and interpreted data. GC, AJO-M, and AP-L drafted the manuscript that was critically revised by the remaining authors for important intellectual content. AJO-M and AP-L had full access to all the data in the study and taken the responsibility for the integrity of the data and the accuracy of the data analysis. All authors approved the final version to be published and agree to be accountable for all aspects of the work in ensuring that questions related to the accuracy or integrity of any part of the work are appropriately investigated and resolved.

## Funding

GC was funded by the Fundação para a Ciência e Tecnologia (FCT; Portugal) through a PhD Scholarship (SFRH/BD/130210/2017). AB was supported by the NIH (NS114405-02, MH120441-01). AJO-M was funded by the FCT (Portugal) through a Junior Research and Career Development Award from the Harvard Medical School—Portugal Program (HMSP-ICJ/0020/2011). GC and AJO-M were supported by grant PTDC/MED-NEU/31331/2017, and AJO-M by grant PTDC/MED-NEU/30302/2017, funded by national funds from FCT/MCTES and co-funded by FEDER, under the Partnership Agreement Lisboa 2020—Programa Operacional Regional de Lisboa. AJO-M was also funded by a Starting Grant from the European Research Council under the European Union's Horizon 2020 research and innovation program (Grant Agreement No. 950357).

## Author Disclaimer

The content of this study is solely the responsibility of the authors and does not necessarily represent the official views of the Fundação para a Ciência e Tecnologia, National Institutes of Health, European Research Council, and Harvard University or its affiliated academic healthcare centers.

## Conflict of Interest

AJO-M was the national coordinator for Portugal of a non-interventional study (EDMS-ERI-143085581, 4.0) to characterize a Treatment-Resistant Depression Cohort in Europe, sponsored by Janssen-Cilag, Ltd (2019–2020), is the recipient of a grant from Schuhfried GmBH for norming and validation of cognitive tests, and is the national coordinator for Portugal of trials of psilocybin therapy for treatment-resistant depression, sponsored by Compass Pathways, Ltd (EudraCT number 2017-003288-36), and of esketamine for treatment-resistant depression, sponsored by the Janssen-Cilag, Ltd (EudraCT NUMBER: 2019-002992-33). AP-L is a co-founder of Linus Health and TI Solutions AG; serves on the scientific advisory boards for Starlab Neuroscience, Magstim Inc., Radiant Hearts, and MedRhythms; and is listed as an inventor on several issued and pending patents on the real-time integration of non-invasive brain stimulation with electroencephalography and magnetic resonance imaging. None of the aforementioned agencies or companies had a role in the design and conduct of the study, in the collection, management, analysis, and interpretation of the data, in the preparation, review, or approval of the manuscript, nor in the decision to submit the manuscript for publication. The remaining authors declare that the research was conducted in the absence of any commercial or financial relationships that could be construed as a potential conflict of interest.

## Publisher's Note

All claims expressed in this article are solely those of the authors and do not necessarily represent those of their affiliated organizations, or those of the publisher, the editors and the reviewers. Any product that may be evaluated in this article, or claim that may be made by its manufacturer, is not guaranteed or endorsed by the publisher.
